# ERO1α promotes hypoxic tumor progression and is associated with poor prognosis in pancreatic cancer

**DOI:** 10.18632/oncotarget.27235

**Published:** 2019-10-15

**Authors:** Nikhil Gupta, Jung Eun Park, Wilford Tse, Jee Keem Low, Oi Lian Kon, Neil McCarthy, Siu Kwan Sze

**Affiliations:** ^1^School of Biological Sciences, Nanyang Technological University, Singapore; ^2^Department of Surgery, Tan Tock Seng Hospital, Singapore; ^3^National Cancer Centre Singapore, Division of Medical Sciences, Singapore; ^4^Centre for Immunobiology, The Blizard Institute, Bart’s and The London School of Medicine and Dentistry, Queen Mary University of London, United Kingdom

**Keywords:** pancreatic cancer, hypoxia tumor, ERO1α, SILAC, proteomics

## Abstract

Pancreatic cancer is a leading cause of mortality worldwide due to the difficulty of detecting early-stage disease and our poor understanding of the mediators that drive progression of hypoxic solid tumors. We therefore used a heavy isotope ‘pulse/trace’ proteomic approach to determine how hypoxia (Hx) alters pancreatic tumor expression of proteins that confer treatment resistance, promote metastasis, and suppress host immunity. Using this method, we identified that hypoxia stress stimulates pancreatic cancer cells to rapidly translate proteins that enhance metastasis (NOTCH2, NCS1, CD151, NUSAP1), treatment resistance (ABCB6), immune suppression (NFIL3, WDR4), angiogenesis (ANGPT4, ERO1α, FOS), alter cell metabolic activity (HK2, ENO2), and mediate growth-promoting cytokine responses (CLK3, ANGPTL4). Database mining confirmed that elevated gene expression of these hypoxia-induced mediators is significantly associated with poor patient survival in various stages of pancreatic cancer. Among these proteins, the oxidoreductase enzyme ERO1α was highly sensitive to induction by hypoxia stress across a range of different pancreatic cancer cell lines and was associated with particularly poor prognosis in human patients. Consistent with these data, genetic deletion of ERO1α substantially reduced growth rates and colony formation by pancreatic cancer cells when assessed in a series of functional assays *in vitro*. Accordingly, when transferred into a mouse xenograft model, ERO1α-deficient tumor cells exhibited severe growth restriction and negligible disease progression *in vivo*. Together, these data indicate that ERO1α is potential prognostic biomarker and novel drug target for pancreatic cancer therapy.

## INTRODUCTION

Pancreatic cancer is associated with &10% patient survival within just 5-years of diagnosis, reflecting a mortality rate approximately double that of other major cancer types [1–6]. Pancreatic Ductal Adenocarcinoma (PDA) is the most common form of pancreatic malignancy but is typically diagnosed only in late-stage disease, hence both the incidence and deaths attributable to PDA continue to increase [7, 8]. At present, there is no clinical procedure that can accurately detect early asymptomatic PDA, largely due to the lack of specific biomarkers of disease [9, 10], hence there is an urgent need for better predictors of tumor development and progression in this patient group with extremely poor prognosis.

Hypoxia frequently affects solid tumors that outgrow their local supplies of oxygen and nutrients. Previous studies have reported that the average pO_2_ of a developing tumor is just 0–5.3 mmHg, whereas the pO_2_ of adjacent healthy tissues ranges between 9.2 and 92.7 mmHg [11], indicating that hypoxia is a major influence on the biology of developing cancers. In particular, hypoxia-inducible factor (HIF-1α) is a key regulator of cellular responses to low-oxygen stress and appears to play a critical role in mediating tumor survival and rate of progression [12–15]*.* In part, this is achieved via HIF-1α induction of pro-angiogenic mediators such as vascular endothelial growth factor (VEGF) [16–18], and transcription factors including Twist, Snail, and ZEB1 that promote epithelial-mesenchymal transition (EMT) [19, 20]. Together, these effects significantly enhance neo-vascularisation of the tumor site, promote tissue invasion/metastasis, and increase chemotherapy resistance in many epithelial cancers. In pancreatic tumors, HIF-1α expression in a CD133+ stem cell-like population has been shown to promote EMT [21], and mutations in HIF-1α itself are key drivers of a variety of cancer types including PDA [22]*.* These data are consistent with the emerging consensus that hypoxia-induced metabolic reprogramming is a hallmark feature of solid tumors including PDA [23], and acts as a key driver of epigenetic changes that promote early invasion and metastasis [24–26], in addition to promoting immunosuppressive phenotypes that limit the effectiveness of many cancer therapies [27, 28]. Despite substantial research progress, the molecular mechanisms that underpin hypoxia-induced effects on tumor development remain poorly understood, hence pancreatic cancer prognosis has failed to improve significantly for many years and treatment options for this disease remain extremely limited.

In the current study, we sought to determine the molecular basis of hypoxia effects on pancreatic cancer progression by using a pSILAC proteomic method (pulsed Stable Isotope Labelling of Amino acid in Cell culture) which facilitates analysis of how environmental factors impact on *de novo* protein synthesis via LC-MS/MS-based quantitation [29, 30]. Using this approach, we investigated how the repertoire of proteins being actively translated by PDA cells is modified by nutrient starvation and hypoxia stresses, before then comparing these data with patient survival statistics and pancreatic cancer gene expression profiles in publically available databases (PRECOG and GEO). These analyses revealed that the oxidoreductase enzyme ERO1α is actively translated by PDA tumors under both hypoxic and serum-free conditions, while also constituting a highly expressed gene associated with poor patient survival in both PRECOG and GEO datasets. Accordingly, genetic deletion of ERO1α inhibited pancreatic tumor proliferation, colony formation, and cellular ROS production *in vitro*, while cell transfer into a mouse xenograft model confirmed a profound reduction in tumor development *in vivo*. Together, these data indicate that hypoxia-induced enzyme ERO1α is a novel therapeutic target in pancreatic cancer and a potential biomarker for enabling earlier disease detection and better prediction of clinical course in human patients.

## RESULTS

### Hypoxia induces dynamic changes in pancreatic cancer cell translational activity

**Figure 1:**
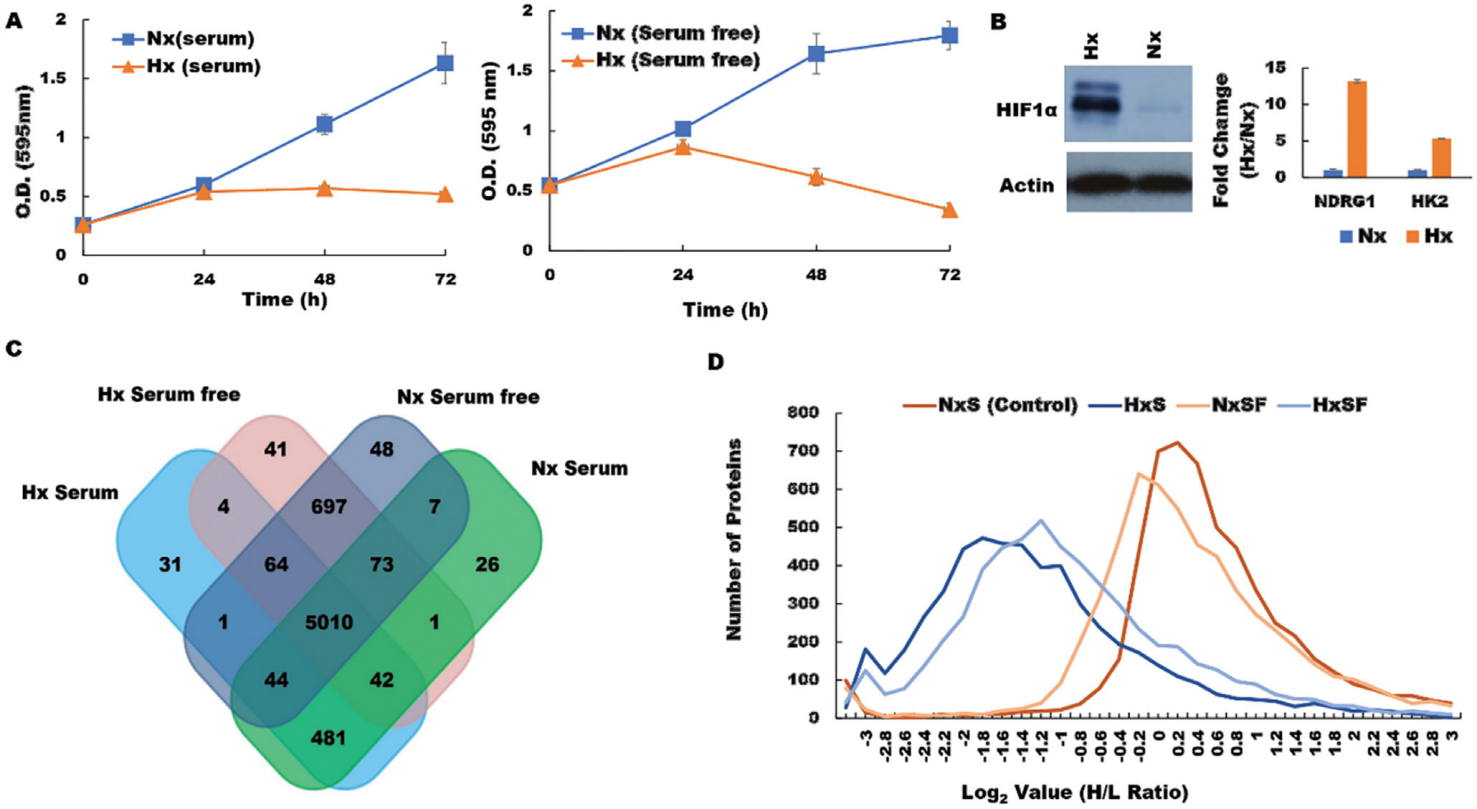
Hypoxia modifies the pancreatic cancer cell proteome under both serum-replete (S) and serum-free conditions (SF). (**A**) PDA cell proliferation curve during culture in S and SF conditions confirming growth restriction during hypoxia. (**B**) Western blot of HIF-1α protein and RT-PCR assessment of fold change (Hx/Nx) in hypoxia markers NDRG1 and HK2. (**C**) the number of proteins identified in PDA cells after culture under the different conditions indicated. (**D**) Analysis of protein distribution against log_2_ H/L ratio for the S and SF culture conditions during normoxia/hypoxia revealed that the majority of these proteins are downregulated during hypoxia. Nx, normoxia; Hx, hypoxia; S, serum/serum replete; SF, serum-free; H/L, heavy/light chain ratio.

The molecular mechanisms by which hypoxia favors pancreatic cancer progression and therapy resistance remain poorly understood. We hypothesized that hypoxic cancer cells co-opt normal metabolic pathways to force translation of proteins that support growth in unfavorable local conditions. To test this concept, we used a pSILAC proteomic approach to investigate how the pancreatic tumor cell proteome is modified in response to a hypoxic microenvironment. To mimic tumor hypoxia *in vivo*, we first cultured pancreatic ductal adenocarcinoma (PDA) cells under low-oxygen conditions *in vitro*, either in the presence or absence of serum, and then compared viability with cells maintained on normal oxygenation (normoxia; Nx) throughout. When assessed in MTT assays, PDA cells displayed stable growth rates over a 24 h culture period irrespective of serum supplementation, and cell viability remained essentially unchanged thereafter. In contrast, PDA cells subjected to low-oxygen conditions displayed reduced proliferation after just 24 h culture (Figure 1A), while substantially upregulating the characteristic hypoxia markers HIF-1α, hexokinase 2 (HK2) and N-Myc Downregulated protein 1 (NDRG1) (Figure 1B). These data confirmed that our model system reproduces the oxygen-deprived state encountered by solid pancreatic tumors *in vivo* and replicates their associated cellular responses *in vitro* [31, 32]. We therefore proceeded to culture PDA cells with light-medium (^12^C_6_, ^14^N_2_-L-Lysine, ^12^C_6_, ^14^N_2_-L-Arginine) before switching to heavy medium (^13^C_6_, ^15^N_2_-D-Lysine, ^13^C_6_, ^15^N_2_-D-Arginine) and subjecting the cells to 24 h culture under normoxia (Nx) or hypoxia (Hx) in the presence or absence of serum. Upon completion of culture, cellular proteins were extracted and analysed by LC-MS/MS in order to identify molecules that incorporated heavy isotope-labelled Lys and Arg (synthesized *de novo* in response to hypoxia stress) and differentiate these from ‘light’ proteins derived from the pre-existing pool within the tumor cells (*n* = 2 biological replicates; Supplementary Data 1). Adjusted *p* values were generated by PD 2.2 software analysis of the pSILAC data. We observed that PDA cells expressed a wide range of different proteins after culture, whether conducted in normoxia under serum replete (5684) or serum-free conditions (5944), or following hypoxia with serum provided (5932), or withheld for the duration (5677) (Figure 1C). Unsurprisingly, the majority of these proteins were derived from a common pool of shared molecules (*n* = 5010), but a substantial fraction were specifically modulated in response to changing microenvironmental conditions (threshold >0.6 log_2_ Fold Change in H/L ratio) (Figure 1D). These candidate mediators of the tumor response to hypoxia stress were subsequently screened and cross-validated by RT-qPCR and western blot in order to verify the pSILAC results. Distribution analyses revealed that most of these proteins were substantially downregulated during hypoxia stress, regardless of serum provision, including several key mediators of cell translation, metabolism, and mRNA maturation processes (Supplementary Figure 1).

**Figure 2:**
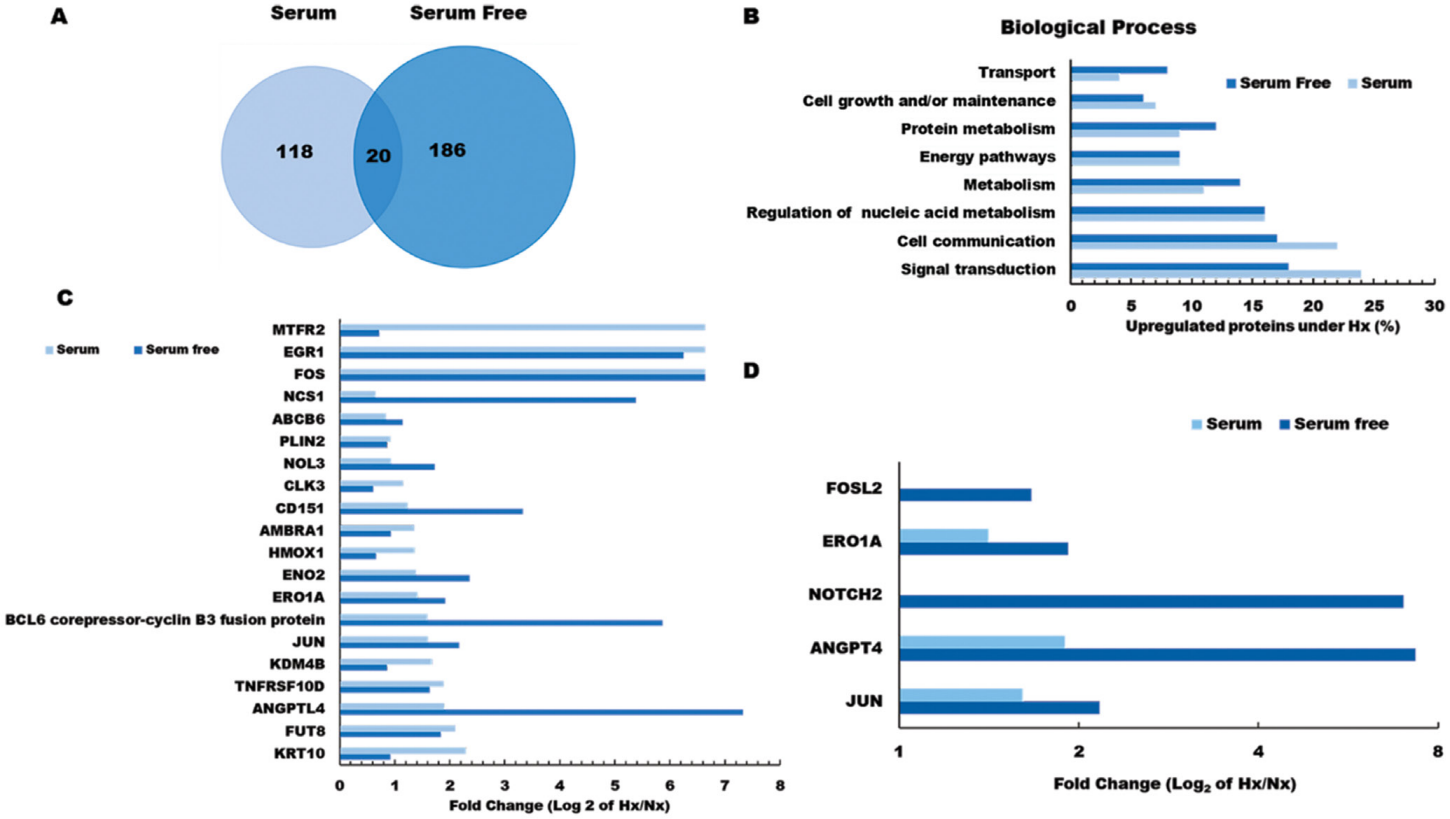
Gene ontology analysis of hypoxia effects on the pancreatic tumor cell proteome. (**A**) Venn diagram showing the number of hypoxia-induced proteins identified as being newly synthesized under S or SF conditions. (**B**) Functional classification of tumor proteins expressed *de novo* in response to hypoxia stress. (**C**) Log_2_ fold change (Hx/Nx) in actively translating proteins detected in either S or SF culture conditions, and (**D**) Log_2_ fold change (Hx/Nx) in angiogenic factor expression comparing S and SF culture conditions. Nx, normoxia; Hx, hypoxia; S, serum/serum replete; SF, serum-free; H/L, heavy/light chain ratio.

Among the proteins induced by pancreatic tumor hypoxia (high Hx/Nx ratios), we observed that 206 were actively synthesized under serum-free conditions, 138 were translated in the presence of serum, and 20 were actively expressed in both conditions (Figure 2A). Comparisons between these groups based on functional classification revealed that the majority of hypoxia-sensitive proteins were mediators of signaling pathways/communication and cell growth, but we also detected marked hypoxia effects on key regulators of protein and nucleic acid metabolism. Several proteins already known to promote tumor metastasis (NOTCH2, NCS1, NUSAP1, CD151), were increased more than 10-fold under hypoxic conditions, further demonstrating the potent effects of oxygen restriction on cancer cell biology. In addition, we observed that hypoxic culture stimulated tumor cell upregulation of multiple proteins that have previously been implicated in host immune suppression (NFIL3, WDR4) and tumor drug resistance (ABCB6), suggesting that these mediators may contribute to poor survival rates among PDA patients.

Further analysis indicated that several critical mediators of cellular transport and metabolic activity were more potently induced by hypoxia under serum-free conditions (Figure 2B and Supplementary Figure 2 and Supplementary Data 2), indicating that nutrient availability plays a major role in determining how oxygen restriction modifies pancreatic tumor biology. Among the 20 proteins induced by hypoxia irrespective of serum presence/absence were several well-established mediators of cancer progression, including key oncogenic transcription factors FOS and JUN, as well as the pro-angiogenic factor ANGPTL4 which is directly involved in neo-vascularization events (Figure 2C). Several other proteins in this group also displayed greater induction under serum-free conditions (NOL3, NCS1, CD151), suggesting synergistic effects of hypoxia and nutrient deprivation on pancreatic cancer cell development and angiogenic activity (Figure 2D). To confirm these findings, gene expression levels were validated by quantitative PCR analyses, which were highly consistent with the pSILAC proteomic data (see Supplementary Figure 2). Having identified a range of hypoxia-induced proteins that are expressed by pancreatic cancer cells *in vitro*, it remained unclear which of these mediators exerted the greatest impact on malignant disease progression *in vivo*. We therefore proceeded to investigate whether hypoxia-induced changes in pancreatic cancer cell proteome impact on disease progression and modify patient outcome using real-world clinical data.

### Oxygen restriction induces pancreatic cancer cell expression of metabolic mediators that predict poor clinical outcome

**Figure 3:**
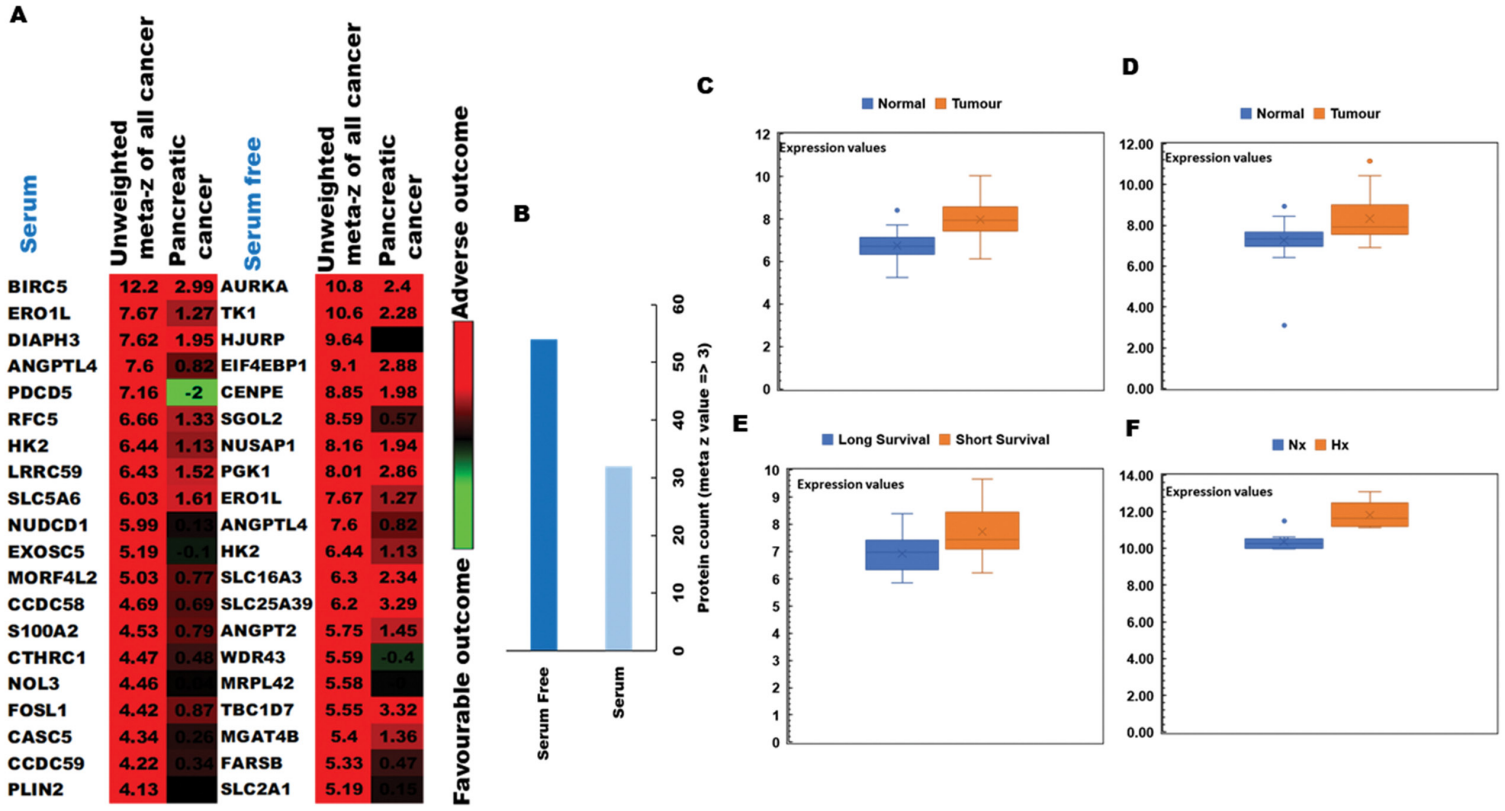
PRECOG analysis of the pancreatic tumor proteome and correlations with patient outcomes. (**A**) Top 20 genes associated with poor clinical prognosis in cancer patients and identified in our pSILAC dataset of hypoxia-induced proteins (high meta *z* values indicating proteins most strongly associated with adverse [red] or favourable [green] survival data) (**B**) Protein count in serum-replete (S) and serum-free (SF) conditions, indicating that markers associated with poor prognosis are more strongly induced by hypoxia (meta *z* value >3) during SF culture. (**C**) Comparison of ERO1α expression levels between healthy tissues and disease samples in relevant GEO datasets: GSE28735; 45 pancreatic tumor samples and 45 matching normal samples (**D**) GSE15471; 78 pancreatic cancer and normal tissue samples (**E**) GSE78229; 50 pancreatic tissue samples grouped by survival duration, and (**F**) GSE67549; *n* = 9 pancreatic cancer cell lines either cultured in Nx or subjected to Hx. Database selection criteria are displayed together with an overview of the 4 studies selected in Supplementary Table 2. Nx, normoxia; Hx, hypoxia; S, serum/serum replete; SF, serum-free; H/L, heavy/light chain ratio.

**Figure 4:**
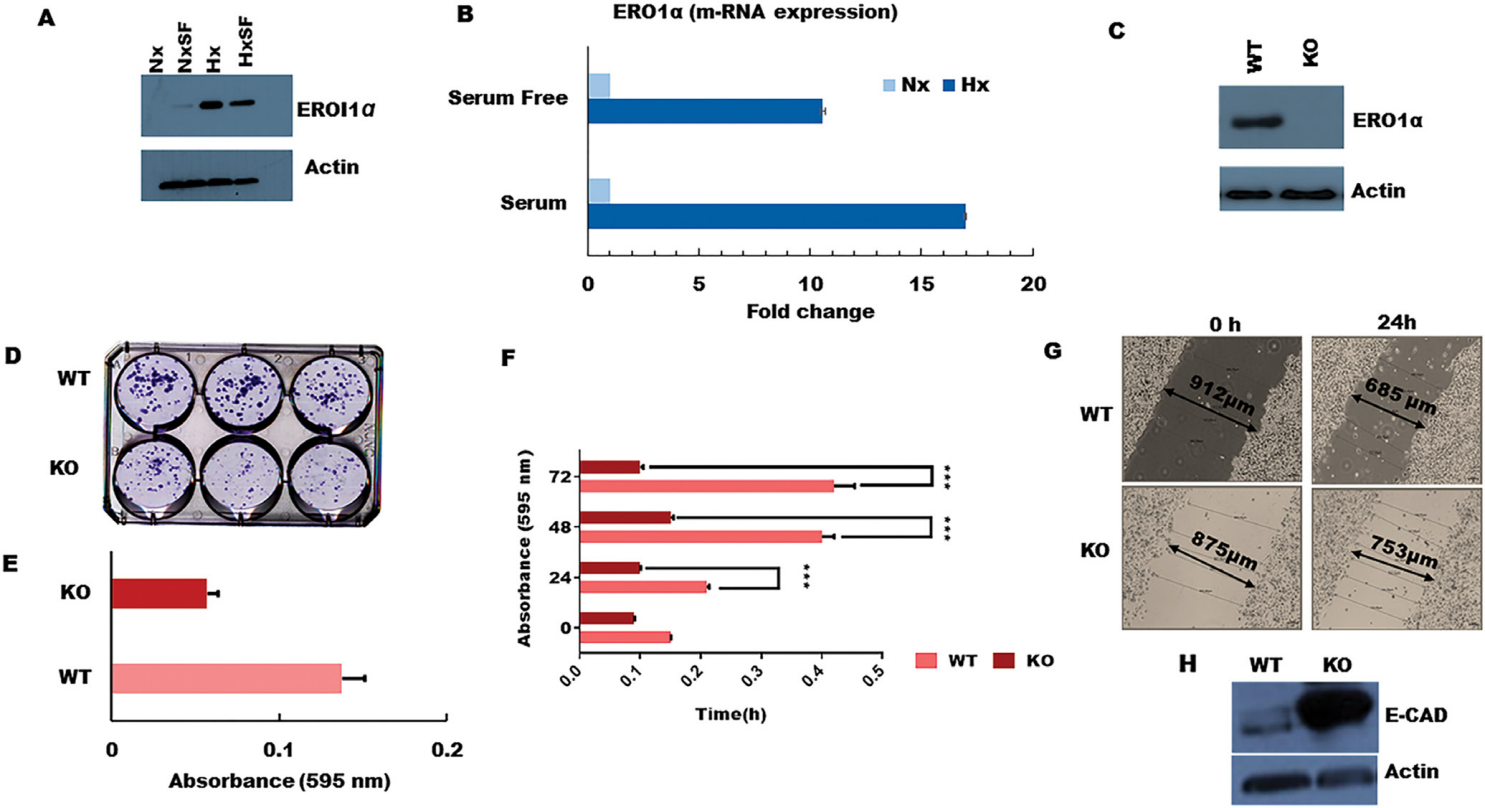
ERO1α deletion reduces PDA tumor cell growth, colony formation, and migratory potential *in vitro*. (**A**) Western blot confirming that ERO1α protein is highly expressed during tumor hypoxia in both S and SF conditions, consistent with the pSILAC data and patient gene expression profiles in the PRECOG and GEO datasets. (**B**) ERO1α mRNA expression levels as assessed using qPCR. (**C**) Western blot analysis of ERO1α expression in WT and ERO1α-KO PDA cells. (**D**, **E**) Colony formation potential of PDA cells comparing WT and ERO1α-KO clones. (**F**) Cell proliferation curves of WT and ERO1α-KO clones in hypoxic MTT assays. (**G**) Wound-healing assay performed using the indicated cell clones and culture conditions, demonstrating the reduced migratory potential of ERO1α-KO tumor cells. (**H**) Western blot analysis of the archetypal EMT suppressor protein E-cadherin, which was overexpressed in ERO1α-KO PDA cells. ^***^ *P* & 0.001. Nx, normoxia; Hx, hypoxia; S, serum/serum replete; SF, serum-free; NxSF, normoxia serum-free; HxSF, hypoxia serum-free; H/L, heavy/light chain ratio; WT, wild type; KO, knockout (ERO1α^- -^).

To determine the clinical relevance of hypoxia-induced changes in protein expression by PDA cells, we conducted ‘PREdiction of clinical outcome from genomic profiles’ (PRECOG) analysis to identify possible correlations of our proteomic results with patient survival data. [28] Hypoxia-induced proteins were first integrated into PRECOG and meta z scores calculated to determine the confidence level of associations with adverse or favorable clinical outcomes (Figure 3A). Using this approach, we observed that several proteins associated with poor prognosis were more strongly induced by hypoxia during serum starvation (Figure 3B) and that meta z scores for the pancreatic cancer dataset were generally higher in serum-free relative to serum-replete conditions. Among these were several proteins for which high gene expression levels were strongly associated with poor patient prognosis, suggesting a likely role in hypoxia-driven PDA progression. In particular, the oxidoreductase protein ERO1α displayed an unweighted meta z score of 7.67 for all cancer types, thus strongly implicating this enzyme as a key determinant of poor clinical outcome in human pancreatic cancer. These data were then verified by qRT-PCR and western blot analyses in both human MIAPaCa-2 and mouse TGP47 pancreatic cancer cells, which confirmed that PDA cells express ERO1α at levels consistent with our proteomic results (Figure 4A and 4B and Supplementary Figure 3). Indeed, further analysis of ERO1α expression patterns in relevant GEO databases, [33] enabled us to confirm that a range of different pancreatic cancer cell lines strongly upregulate ERO1α under hypoxic conditions (Figure 3F). Mining of additional publicly available datasets (Supplementary Table 2) revealed that this enzyme is also expressed at significantly higher levels in pancreatic tumor samples compared with adjacent healthy tissue (Figure 3C and 3D), in clear association with adverse patient outcomes (Figure 3E). Together, these data strongly indicate that under hypoxic conditions, pancreatic tumor cells significantly upregulate the enzyme ERO1α, which is a critical determinant of pancreatic cancer patient survival. While ERO1α plays a well-established role mediating disulfide bond formation in a range of different secreted and membrane proteins, the survival advantages this confers on PDA cells and the mechanisms involved remained unknown.

### ERO1α mediates pancreatic cancer cell growth, ROS production, and tumorigenicity *in vivo*

To better understand the role of ERO1α in pancreatic cancer progression, we next deleted the corresponding gene using CRISPR/Cas genome editing technology and assessed the impact on tumor cell function both *in vitro* and *in vivo*. For this, we selected a sgRNA with the highest ‘on target’ score, which was directed against exon 7 of human ERO1α and predicted to induce a frameshift mutation that generates non-functional/truncated gene products. We then infected human pancreatic cancer cells with Cas9-expressing lentiviral vectors in order to disrupt the ERO1α gene and used Sanger sequencing to verify mutation at the predicted site. Accordingly, western blot analysis of ERO1α expression in the treated cells confirmed that our gene disruption strategy achieved complete knockout of ERO1α protein (Figure 4C), so we proceeded to test whether pancreatic tumor cell proliferation rates differed between WT and ERO1α-KO clones. Unlike their WT counterparts, ERO1α-KO tumors displayed little to no growth over a 24–72 h culture period (Figure 4F), and colony formation was reduced by as much as 50% (Figure 4D and 4E), suggesting that ERO1α deletion significantly reduces the replicative capacity of pancreatic cancer cells. Furthermore, when assessed in a series of wound healing assays, we observed that ERO1α-KO tumor cells displayed impaired ability to close the injury due to migration rates being reduced by half relative to ERO1α-competent tumor cells (Figure 4G). Given these marked defects in cell motility, we next assessed tumor cell expression of cadherins, which play a central role in restricting cancer cell migration and tissue invasion. When analyzed by western blot, key EMT suppressor E-cadherin was found to be highly expressed in ERO1α-KO tumor cells, perhaps explaining their reduced migratory potential in our functional assays (Figure 4H).

**Figure 5:**
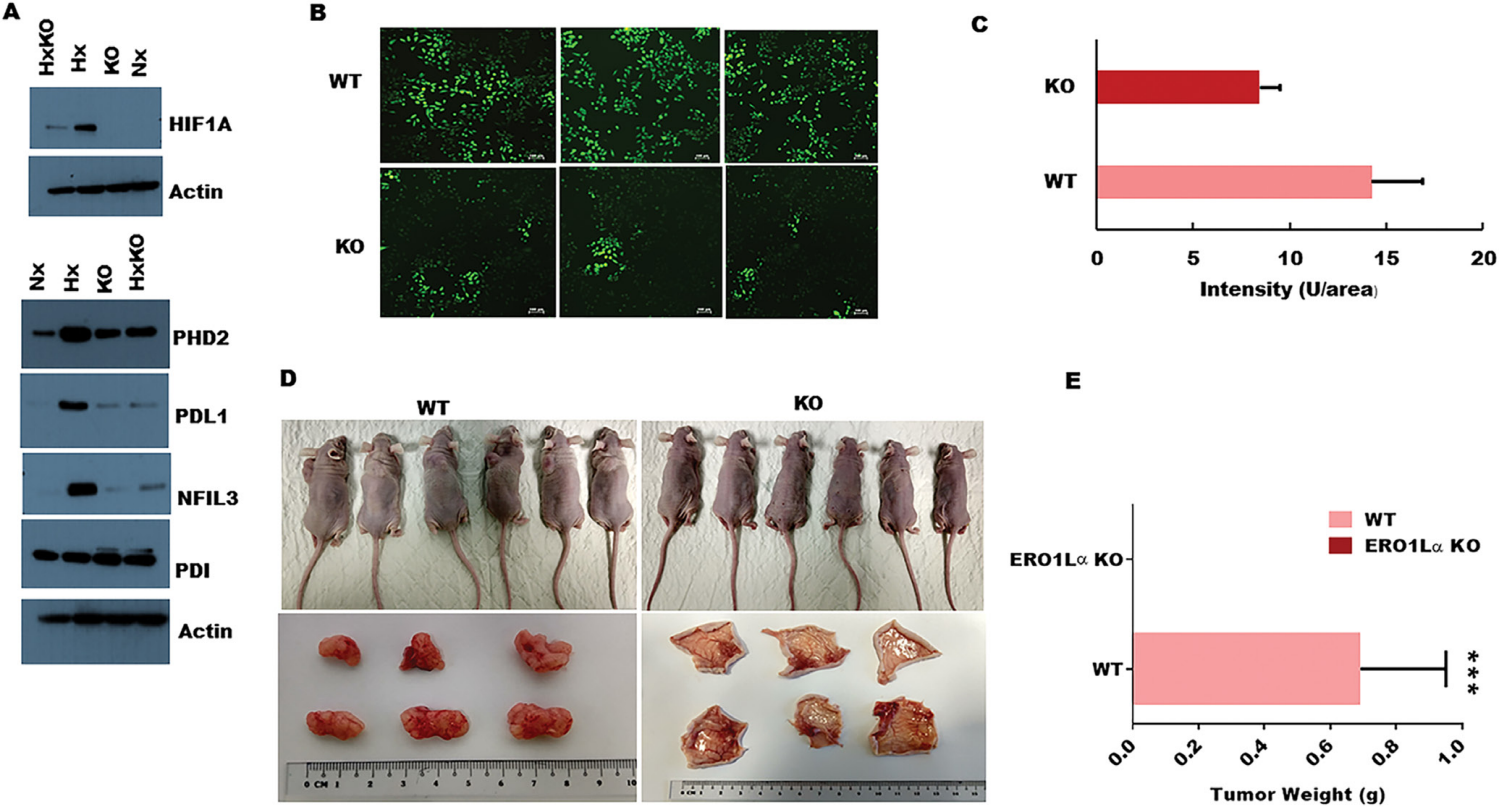
Impact of ERO1α deletion on oncogenic protein expression, ROS production, and xenograft tumor progression *in vivo*. (**A**) Western blot analysis of ERO1α-regulated proteins comparing WT with ERO1α-KO tumor cells. (**B**) DCFDA assay with representative fluorescence images of WT and ERO1α-KO PDA cells showing a significant reduction in ROS generation in ERO1α-deficient tumors. (**C**) Fluorescence intensity was measured by microplate reader confirming significant ROS impairment in ERO1α-KO clones (**D**) Representative images of xenografted mouse tumor injection sites before and 28 days after inoculation with the indicated cancer cells. (**E**) Tumors were excised and weighed; (mean ± s.d., *n* = 6/group. ^***^ *P* & .001 (student *t*-test). WT, wild type; Hx, hypoxia; KO, knockout (ERO1α^- -^); HxKO, hypoxia knockout (ERO1α^- -^).

Having detected a clear impact of ERO1α deletion on PDA cell expression of a key protein regulator of tumor development, we next investigated whether ERO1α was also required to induce other critical mediators of disease progression. In particular, HIF-1α is a critical regulator of many oncogenic pathways known to be triggered by hypoxia, and our western blot analyses revealed a significant deficit in expression of this transcription factor in ERO1α-KO cells (Figure 5A) [34]. Furthermore, in the absence of ERO1α, we observed that PDA cells also displayed substantially reduced expression of the immune inhibitory molecule PD-L1, which is typically upregulated in many different cancer types (Figure 5A). Given that HIF-1α expression is known to be controlled by reactive oxygen species (ROS), we next assessed whether the generation of these mediators might represent an important mechanism by which ERO1α influences pancreatic cancer progression. Indeed, previous work has identified that ERO1α oxidizes PDI which in-turn catalyzes the formation of disulfide bonds in folding proteins, while simultaneously generating intracellular hydrogen peroxide (H_2_O_2_) that can mutate DNA and trigger oncogenic events. When assessed in fluorescence-based DCDFA assays, we observed that PDA cells lacking ERO1α displayed substantially reduced ability to generate ROS, exhibiting only 66% of the ROS capacity detected in WT tumor cells (Figure 5B and 5C). Together, these data confirmed that ERO1α-deficient PDA cells exhibit impaired tumor development *in vitro*, so we next performed xenograft experiments in Ncr-nude mice to determine whether these cells also displayed reduced oncogenic potential *in vivo*. When administered by subcutaneous (s.c) injection, ERO1α-competent tumors developed readily within just 2 weeks of cell transfer, whereas ERO1α-deficient cancer cells failed to drive tumor formation in recipient animals (*n* = 6 per group). Indeed, when skin was excised from the injection sites we were unable to detect significant tumor development in any mice that had received ERO1α-KO PDA cells (Figure 5D and 5E). These data confirm that in the absence of ERO1α, pancreatic tumor cells undergo regression rather than progression *in vivo*.

## DISCUSSION

Low oxygen availability in solid tumors is now well established as a crucial factor driving malignant progression, promoting treatment resistance, and conferring poor clinical outcome in pancreatic cancer. [35] Since the molecular basis of these effects remains poorly defined, we used a pSILAC proteomic approach to identify hypoxia-sensitive proteins expressed by pancreatic cancer cells when subjected to oxygen restriction *in vitro* either in the presence or absence of serum supplementation (mimicking restricted blood/nutrient supply *in vivo*). Using this model, we detected rapid induction of several established biomarkers of tumor hypoxia and observed that active protein translation was broadly downregulated in the oxygen-deprived microenvironment. However, we also identified that hypoxic stress induces pancreatic tumor cell expression of the oxidoreductase enzyme ERO1α, which displayed a clear association with poor survival statistics mined from patient gene expression databases.

Hypoxia is known to impede the growth of both tumors and healthy cell types, but variable severity and duration of hypoxia can induce a range of different effects that have been shown to increase cancer cell viability, therapy resistance, and metastatic potential [34, 36, 37]. While tumor translational activity was generally downregulated during low-oxygen stress in our assays, we also detected a group of proteins that were instead selectively induced by hypoxia (206 in serum-free cultures, 138 under serum-replete conditions, and 20 that were actively synthesized in both settings). The relatively high number of tumor proteins upregulated in serum-free culture might indicate translation of molecules that support cancer cell survival under adverse conditions. In support of this concept, the profile of proteins induced in the presence/absence of serum displayed divergent functional profiles, with serum-replete culture favoring mediators of cell communication and signal transduction, whereas serum-free conditions induced regulators of cellular transport and metabolic processes. Hypoxia stress also induced several proteins that have been shown to increase metastasis, promote immune suppression, and enhance treatment resistance in multiple different cancers. One such highly-upregulated (Hx/Nx >10) molecule in our dataset was the calcium-binding protein NCS1 (neuronal calcium sensor 1) which performs a variety of different functions in normal cell biology but has also been shown to promote metastasis in breast cancer. [38] Furthermore, our data indicated that hypoxic tumors subjected to serum starvation displayed greater induction of the anti-apoptotic protein NOL3, key mediator of tumor migration/invasion CD151, and angiogenic factor ANGPTL4 which supports tumor neovascularization. Together, these data indicate that hypoxia effects on pancreatic tumor development are strongly influenced by the nutrient status of the constituent cancer cells, which subsequently upregulate key mediators of cell survival, dissemination, and angiogenic processes.

In order to determine the clinical relevance of hypoxia-induced protein translational activity in pancreatic cancer cells, we integrated our data with cancer patient survival statistics available via public gene expression databases. Using the ‘PRECOG’ tool to identify associations between gene expression levels and clinical outcomes, we were able to identify which hypoxia-triggered proteins exert the greatest influence on cancer patient prognosis. Intriguingly, proteins associated with poor prognosis were more actively translated by hypoxic tumors in serum-free cultures than in serum-replete conditions, further indicating that nutrient availability is a major determinant of how hypoxia impacts cancer progression. Further screening of these proteins revealed that ERO1α was associated with a particularly poor prognosis and displayed a high meta z score irrespective of serum availability. We therefore analyzed the expression of ERO1α in relevant GEO datasets, which confirmed that ERO1α is induced by hypoxia stress across a range of pancreatic cell lines. These analyses also indicated that ERO1α is expressed at elevated levels in pancreatic cancers relative to adjacent healthy tissues from the same patient, and that high tumor expression of this enzyme is significantly correlated with short survival duration.

Previous studies have implicated ERO1α in a range of different cancer types, with current evidence suggesting potential roles in cancer cell invasion of healthy tissues, acquisition of chemoresistant properties, and promotion of angiogenesis [39]. Recent data have also suggested that ERO1α-mediated protein folding is required for the generation of myeloid-derived suppressor cells that protect tumors against immune destruction [40]. In order to test the function of ERO1α in pancreatic cancer cells, we used a CRISPR/Cas9 approach to delete the corresponding gene and assessed the impact on oncogenic potential in a range of different assays. While ERO1α-KO pancreatic cancer cells were morphologically indistinguishable from their wild-type counterparts, tumor proliferation and colony-forming potential were significantly reduced in the absence of this enzyme. Similarly, when we assessed the motility of ERO1α-KO tumor cells using *in vitro* wound healing assays, we observed a marked reduction in migratory potential compared with WT clones. Since ERO1α enzyme activity is known to generate reactive oxygen species (ROS) which directly regulate the hypoxia response through transcription factor HIF-1α, we next assessed whether ROS generation might underpin the influence of ERO1α on pancreatic cancer progression. Using DCFDA assays to quantify ROS levels in hypoxic tumor cells, we observed that ERO1α-KO clones displayed a significant reduction in ROS generation relative to WT clones. Accordingly, when WT or ERO1α-KO cancer cells were injected into BALB/c nu/nu mice, the enzyme-deficient tumors failed to develop whereas wild-type cells were highly tumorigenic *in vivo*. These data are consistent with a previous report that under hypoxic conditions, ERO1α can also support the development of colorectal cancer by mediating formation of disulfide bonds that activate integrin signaling and modulate expression of EMT markers [41]. Further research will now be required to determine whether ERO1α also impacts on other pathways such as PI3K-AktmTOR signalling which are frequently activated in pancreatic cancer and known to modulate cell proliferation, protein production, genomic stability, cellular metabolism, and metastatic potential.

Taken together, these data indicate that a combination of microenvironmental hypoxia and restricted blood/nutrient supply trigger pancreatic cancer cell translation of a range of different proteins that act to increase malignancy and are associated with poor clinical outcome. In particular, the oxidizing enzyme ERO1α is predominantly activated only under abnormal conditions such as hypoxia, which perhaps explains the marked induction of this protein in oxygen-starved tumors. Consistent with this concept, our data confirmed that ERO1α-deficient pancreatic cancer cells displayed negligible ability to form tumors *in vivo*, thus implicating ERO1α as a potential novel target for cancer therapy in human patients. Given that ERO1α function appears less critical under steady-state conditions, it is also possible that therapeutic disruption of this enzyme may succeed in limiting tumor growth and metastasis without significantly damaging healthy cells and tissues. Further investigations will now be required to assess this possibility. Collectively, the findings of this systematic study using pSILAC-based quantitative proteomics reveal that tumor hypoxia induces active translation of a range of critical proteins that are associated with poor prognosis in human cancer patients. Specifically, our data reveal that the oxidoreductase enzyme ERO1α is a major proliferation regulator in pancreatic cancer cells both *in vitro* and *in vivo*, hence this protein may constitute a useful diagnostic marker of tumor progression and possible new therapeutic target in pancreatic malignancy. Further study will now be necessary to understand the precise role of ERO1α within the tumor microenvironment, determine how this enzyme influences ECM remodelling, and assess potential associations with tumor progression via conditional knockout/overexpression in xenograft and genetically engineered mouse models.

## MATERIALS AND METHODS

### Cell culture

Pancreatic cancer cells of human (MIAPaCa-2) or mouse origin (TGP47) were purchased from the American Type Culture Collection (Manassas, VA, USA) and cultured in Dulbecco’s Modified Eagle’s Medium (DMEM) (Biowest, France) or a mixture of DMEM and Ham’s F12 medium (1:1) supplemented with 10% foetal bovine serum (FBS) (Gibco, USA) and 1% Penicillin/Streptomycin (Naclai-Teqsue, Japan) in a humidified incubator at 37°C with 5% CO_2_. For serum starvation experiments, the cells were washed three times in 1×PBS before incubating in FBS-free media for 24 h. For hypoxia experiments, cells were subjected to low-oxygen culture (&0.1% O_2_, 5% CO_2_, 95% N_2_) in a hypoxia chamber for 24 h. Cell lysates were subsequently obtained for protein and gene expression analyses. Cell proliferation was measured using MTT assays as previously described [42].

### pSILAC labeling and proteomic sample preparation

MIAPaCa-2 cells were initially grown for 24 h in ‘light’ culture medium (containing unlabelled 146 mg/l ^12^C_6_, ^14^N_2_-L-Lysine and 84 mg/l ^12^C_6_, ^14^N_2_-L-Arginine), then switched onto ‘heavy’ medium (containing labelled 146 mg/l ^13^C_6_, ^15^N_2_-D-Lysine and 84 mg/l ^13^C_6_, ^15^N_2_-D-Arginine) and cultured for a further 24 h under normoxic or hypoxic conditions in the presence or absence of FBS (*n* = 2 independent biological replicates). Cells were then washed with cold PBS and lysed using 8 M urea buffer containing cOmplete™ EASYpack protease inhibitor cocktail (Sigma Aldrich, USA). In-solution digestion was performed as previously described [26]. Extracted peptides were subjected to fractionation on an XBridge™ BEH C18 column (4.6 × 250 mm; Waters Corporation, Milford, MA, USA) and analyzed by liquid chromatography-tandem mass spectrometry (LC-MS/MS).

Peptides were separated and analyzed on a Dionex Ultimate 3000 RSLCnano system coupled to a Q-Exactive Hybrid Quadrupole-Orbitrap mass spectrometer (Thermo Fisher Scientific Inc, Germany). Separation was performed on a Dionex EASY-Spray 75 µm×10 cm column packed with PepMap C18 3 µm, 100 A° (ThermoFisher Scientific Inc. Germany) using solvent A (0.1% formic acid in 99.9% water) and solvent B (0.1% formic acid in 90% ACN) at flow rate of 300 nl/min with a 60 min gradient. Peptides were subsequently analyzed using the Q-exactive MS with an EASY nanospray source (Thermo Fisher Scientific Inc, Germany) at an electrospray potential of 1.5kV. A full MS scan (350 – 1,600 m/z range) was acquired at a resolution of 70,000 at m/z 200 and a maximum ion accumulation time of 100ms. Dynamic exclusion was set as 15s. The resolution of high energy collisional dissociation (HCD) spectra was set to 17,500 at m/z 200. The automatic gain control settings of the full MS and MS2 scans were 3E6 and 2E5, respectively. The 10 most intense ions above a 2,000-count threshold were selected for HCD fragmentation, with a maximum ion accumulation time of 100ms. An isolation width of 2 was used for MS2. Single and unassigned charged ions were excluded from MS/MS. For HCD, the normalized collision energy was set to 28%. The underfill ratio was defined as 0.2%.

Protein identification and quantitation were performed by processing the raw data from three replicates using Protein Discoverer (PD) software version 2.2 (Thermo Scientific Inc. Germany). The MS/MS spectra were deisotoped and deconvoluted using the MS2 spectrum processor node in PD. Mascot and Sequest HT were used in parallel and the data compared against a protein sequence file from the UniProt human database (downloaded on 06 Feb 2017, 1,586,248 sequences, 61,972,042 residues). For searches using both engines, maximum missed cleavage sites per protein was set at 2, with precursor and fragment ions mass tolerance set at 10ppm and 0.02Da respectively. Carbamidomethylation (C) was set as a fixed/static modification. SILAC_R6 (R)/13C(6), SILAC_K8 (K)/13C(6)15N(2), acetylation (Protein N-term), deamidation (NQ) and Oxidation (M) were set as dynamic/variable modifications in both searches. A protein group list was generated from PD 2.2 software and the list was filtered to exclude contaminating proteins. Only proteins with *q*-value &0.01 (&1% FDR) as determined by percolator and detected in both replicates were used for further analysis. The mass spectrometry proteomics data have been deposited with the ProteomeXchange Consortium via the PRIDE partner repository under dataset identifier PXD014087.

### Western blot

Cells were washed twice in cold PBS and lysed in RIPA buffer (0.1% SDS, 1% NP-40, 1% sodium deoxycholate, 150 mM NaCl, 1 mM EDTA, and 50 Mm Tris-HCl pH 7.4, with cOmplete™ EASYpack protease and phosphatase inhibitors) (Sigma-Aldrich, USA). Cell lysates were subjected to western blotting using the following primary antibodies: anti-ERO1α (CST), anti-HIF-1α (CST), anti-PDL1 (Abcam), anti-NFIL3 (CST), anti-PHD2 (CST), and anti-E-CAD (BD Bioscience). The loading control was beta-actin (1:5000) (EMD Millipore, USA). Proteins bound by each antibody were visualized using SuperSignal™ West Pico Chemiluminescence kit (Thermo Fisher Scientific, USA).

### RNA isolation and qPCR

Total RNA was isolated using Nucleospin RNA II kits (Macherey-Nagel GmbH, Germany). Quantitative PCR (qPCR) reactions were performed using the CFX Connect™ Real-Time PCR Detection System (Bio-Rad Laboratories, Inc.) with KAPA SYBR^®^ FAST qPCR Master mix. Actin was used as an internal control. The primer sequences used for qPCR are provided in Supplementary Table 1.

### Bioinformatic analysis

The pSILAC protocol allows newly synthesized proteins incorporating heavy (H) arginine and lysine to be distinguished from pre-existing proteins that include only light (L) arginine and lysine residues. Change in pSILAC H/L ratio under low-oxygen conditions can therefore be used to determine the impact of hypoxia on tumor cell protein expression activity. Proteins that were actively translated in response to hypoxia either in the presence of serum (HS/NS >1.5) or under serum-free conditions (HSF/NSF >1.5) (*p* &=0.001) were shortlisted for further analysis. Gene ontology and pathway analyses were performed using Functional Annotation (FunRich) [43]. Prediction of Clinical Outcomes from Genomic profiles (PRECOG) and Gene Expression Omnibus (GEO) web tools were used to correlate the pSILAC proteomic data with clinical pancreatic tumor gene expression profiles in order to identify hypoxia-inducible proteins associated with poor prognosis [44, 45]. Hypoxia-induced proteins that also displayed upregulated gene expression in cancer cells as reported in the GEO database were identified as potential targets. These targets were then further analyzed using PRECOG to query associations between gene expression profiles and real-world patient outcomes. Potential targets associated with poor prognosis were identified by PRECOG meta z score. ERO1α displayed one of the highest meta z scores in the dataset and was therefore followed-up in subsequent functional studies. Datasets GSE15471, GSE78229, GSE67549 and GSE28735 reporting the results of pancreatic cancer microarrays in the NCBI GEO database (https://www.ncbi.nlm.nih.gov/geo/) were analyzed to confirm differential ERO1α expression [46–48].

### CRISPR/Cas9 deletion of ERO1α

All CRISPR gRNA were computationally designed using CHOPCHOP V.2 software (Harvard, USA) to calculate ‘on target’ score and predicted Cas9 cleavage efficiency using a range of different algorithms (Benchling, San Francisco, CA, USA). Three gRNA were designed (Supplementary Table 3), and target sequences were cloned into lentiCRISPR v2 plasmids (Addgene plasmid # 52961) as described previously [49].

### Colony formation and wound-healing assays

Colony formation assays were performed as previously described using a total of 200 cells/chamber seeded into six-well plates and cultured in DMEM [50]. To quantify migratory potential, cells were spread and cultured until confluence, then scratched using a 200 µl pipette tip before washing and incubating in fresh serum-free medium. After 24 h culture, scratch dimensions were analyzed using an optical microscope [51].

### Intracellular ROS detection

Intracellular ROS analysis was performed as previously described [52]. Briefly, cells were incubated with 10 µM DCFDA in culture media for 1 h at 30°C before harvesting, then washed twice in ice-cold PBS and examined under a fluorescent microscope (λex = 495 nm λem = 530 nm) to identify green fluorescence generated by ROS production.

### Xenografted tumor development assay

All animal studies were approved and conducted in compliance with the guidelines of the Institutional Animal Care and Use Committee (IACUC) of Nanyang Technological University. Ncr nude mice (males aged 7 weeks) were obtained from InVivos Pte, Ltd (Singapore). For tumor formation studies, mice were administered 3 × 10^6^ MIAPaCa-2-Con cells (*n* = 6) or MIAPaCa-2-ERO1α^-/-^ (KO) cells injected subcutaneously into the neck area and tumor size was monitored by caliper measurement. Mice were euthanized when tumor size exceeded 2000 mm^3^.

### Statistical analysis

Statistical analysis was performed using GraphPad Prism 6.0 software (GraphPad Softwares Inc., La Jolla, CA, USA). Differences between variables were assessed using Student’s paired *t*-test, and *P* & 0.05 was considered statistically significant. FunRich tool provided *p*-values for each ontology enrichment score. GEO datasets and PRECOG tool enabled statistical analysis of overall survival and z scores for the high- and low-risk groups.

## SUPPLEMENTARY MATERIALS






