# Supplementary material for: ERO1α promotes hypoxic tumor progression and is associated with poor prognosis in pancreatic cancer
Source: Oncotarget. 2019 Oct 15;10(57):5970–82. doi: 10.18632/oncotarget.27235 (PMC6800261; doi:10.18632/oncotarget.27235)
Supplement: Supplementary file 1 [file oncotarget-10-5970-s001.pdf]

ERO1α promotes hypoxic tumor progression and is associated with poor prognosis in pancreatic cancer

SUPPLEMENTARY MATERIALS

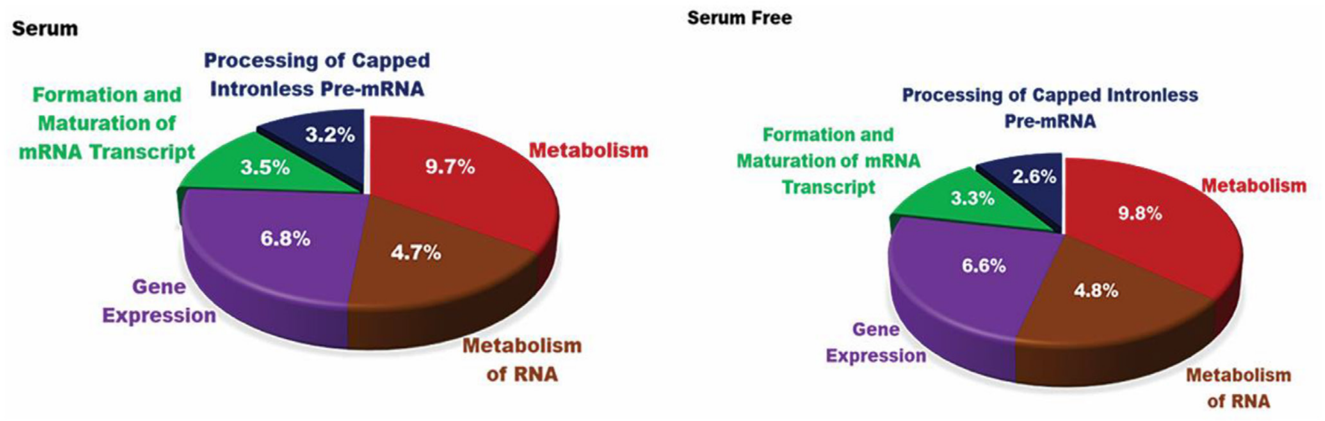

Supplementary Figure 1: Gene ontology analysis of the proteins downregulated by hypoxic tumor cells in either serum-replete or serum-free conditions.

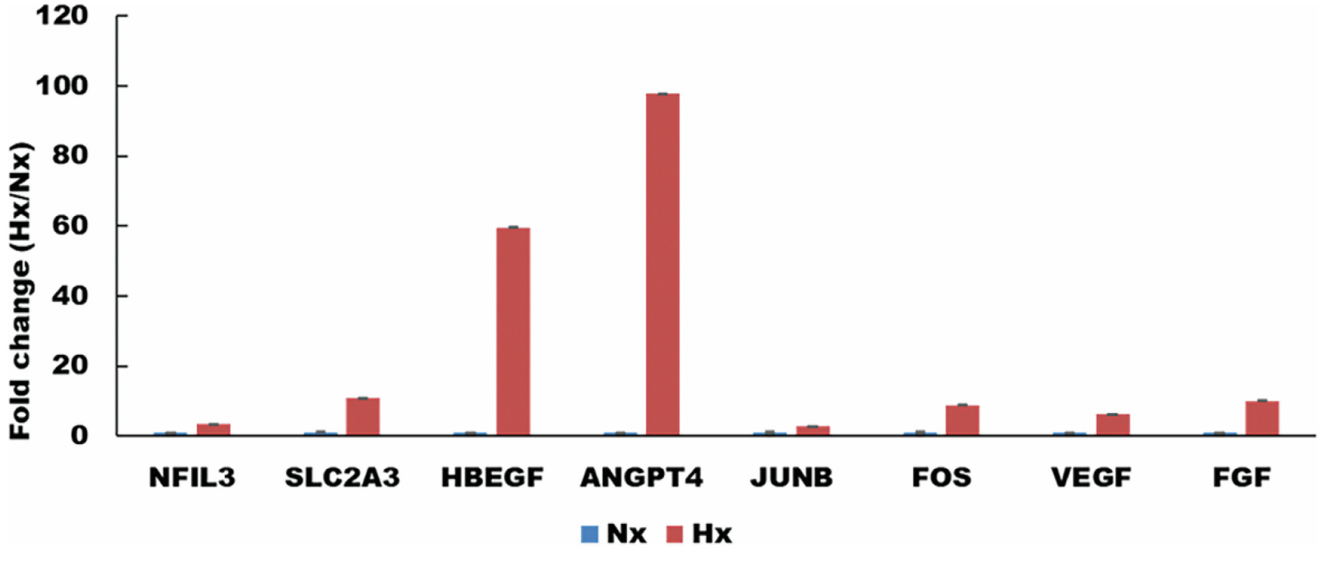

Supplementary Figure 2: qPCR validation of key hypoxia-induced proteins as identified by pSILAC analysis (expressed as Hx/Nx fold-change in gene expression, *n* = 3).

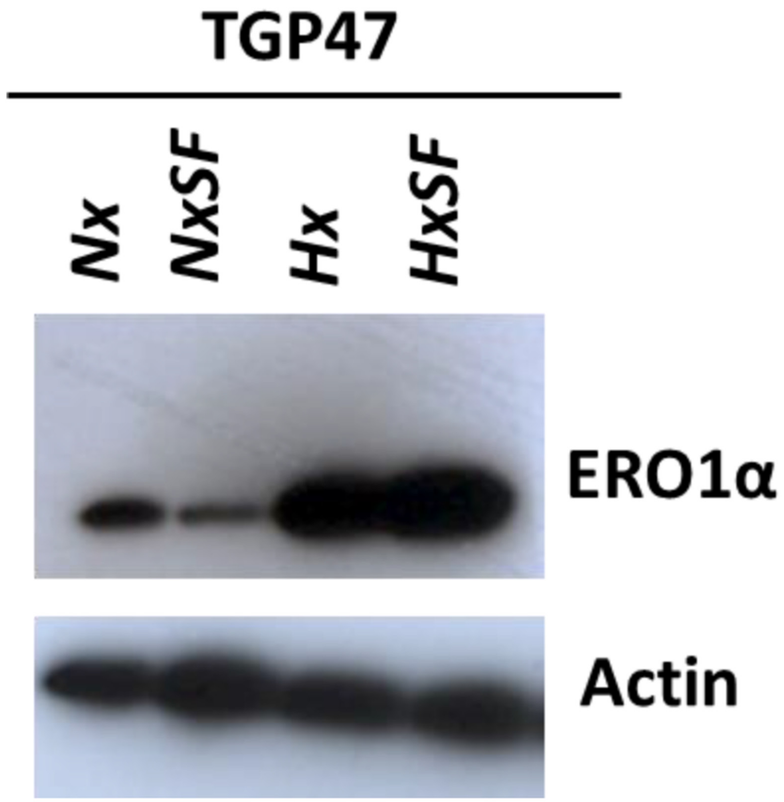

Supplementary Figure 3: Western blot of ERO1α expression in TGP47 mouse pancreatic cancer cells; Nx, normoxia; NxSF, normoxia serum-free; Hx, hypoxia; HxSF, hypoxia serum-free.

Supplementary Data 1: Data directly exported from Proteome Discoverer version 2.2 for pSILAC proteomic analysis of pancreatic cancer cell line (MIAPaCa-2) in serum and serum free condition under hypoxia. See Supplementary_Data_1

Supplementary Data 2: Results from bioinformatic data analysis. See Supplementary_Data_2

Supplementary Table 1: Primer sequences for quantitative PCR

Primer	5′–3′
ERO1α_FW	ATGACATCAGCCAGTGTGGA
ERO1α_RV	CATGCTTGGTCCACTGAAGA
NFIL3_FW	AATGCAGACCGTCAAAAAGG
NFIL3_RV	TTGTTCTTCCCCACACTTCC
HBEGF_FW	GGTGGTGCTGAAGCTCTTTC
HBEGF_RV	GCTTGTGGCTTGGAGGATAA
VEGFA_FW	AAGGAGGAGGGCAGAATCAT
VEGFA_RV	ATCTGCATGGTGATGTTGGA
FGF1_FW	TGCCTCCAGGGAATTACAAG
FGF1_RV	TATAAAAGCCCGTCGGTGTC
ACTIN_FW	GGACTTCGAGCAAGAGATGG
ACTIN_RV	AGCACTGTGTTGGCGTACAG
SLC2A3_FW	ACCGGCTTCCTCATTACCTT
SLC2A3_RV	AGGCTCGATGCTGTTCATCT
ANGPTL4_FW	GCCTATAGCCTGCAGCTCAC
ANGPTL4_RV	AGTACTGGCCGTTGAGGTTG
JUNB_FW	AATGGAGGACACGGACTTTG
JUNB_RV	ATGTCTCTGGCATCCCTACG
FOS_FW	AGAATCCGAAGGGAAAGGAA
FOS_RV	CTTCTCCTTCAGCAGGTTGG
NDRG1_FW	ACAACCCTGAGATGGTGGAG
NDRG1_RV	TGTGGACCACTTCCACGTTA
HK2_FW	TCTATGCCATCCCTGAGGAC
HK2_RV	TCTCTGCCTTCCACTCCACT

Supplementary Table 2: Microarray dataset used for ERO1α expression analysis

S.NO.	GEO accession	Platform	Sample	Cancer Type
1	GSE15471	GPL570	Whole-tissue pancreatic ductal adenocarcinoma	Pancreatic Cancer
2	GSE78229	GPL6244	Pancreatic tumours tissue	Pancreatic Cancer
3	GSE67549	GPL15207	Cancer cell line	Pancreatic Cancer
4	GSE28735	GPL6244	Pancreatic tissue and adjacent normal tissue	Pancreatic Cancer

Supplementary Table 3: ERO1α CRIPSR oligo design

ERO1α CRIPSR oligo design	
**Name**	**Sequence**
**Exon 7 CHOP CHOP (No mismatch)**	
ERO1Lα_Oligo1_LENTI_guide	CACCGGAGCGCTACACTGGTTACAA
ERO1Lα_Oligo2_LENTI_guide	AAACTTGTAACCAGTGTAGCGCTAC
**Exon 1 CHOP CHOP (3 mismatches)**	**Low Efficiency**
ERO1Lα2_Oligo1_LENTI_guide	CACCGCGCGGCTGGGGATTCTTGTT
ERO1Lα2_Oligo2_LENTI_guide	AAACAACAAGAATCCCCAGCCGCGC
**Exon 1 CHOP CHOP (3 mismatches)**	**High Efficiency**
ERO1Lα3_Oligo1_LENTI_guide	CACCGGCCGCGGCCCATTGCAGCTC
ERO1Lα3_Oligo2_LENTI_guide	AAACGAGCTGCAATGGGCCGCGGCC
